# Genomic and epigenomic coordination maintains subgenome transcriptional balance in allotetraploid *Brassica napus*

**DOI:** 10.1093/hr/uhaf266

**Published:** 2025-10-03

**Authors:** Jie Zhou, Meng Ma, Qing Zhang, Shangyan Ni, Hu Zhao, Jing Wen, Jinxiong Shen, Tingdong Fu, Lun Zhao

**Affiliations:** National Key Laboratory of Crop Genetic Improvement, National Engineering Research Center of Rapeseed, Hubei Hongshan Laboratory, Huazhong Agricultural University, Wuhan 430070, China; State Key Laboratory of Cotton Bio-Breeding and Integrated Utilization, Institute of Cotton Research of Chinese Academy of Agricultural Sciences, Anyang Henan 455000, China; College of Agriculture, State Key Laboratory of Crop Stress Adaption and Improvement, Henan University, Kaifeng Henan 475004, China; National Key Laboratory of Crop Genetic Improvement, National Engineering Research Center of Rapeseed, Hubei Hongshan Laboratory, Huazhong Agricultural University, Wuhan 430070, China; National Key Laboratory of Crop Genetic Improvement, National Engineering Research Center of Rapeseed, Hubei Hongshan Laboratory, Huazhong Agricultural University, Wuhan 430070, China; National Key Laboratory of Crop Genetic Improvement, National Engineering Research Center of Rapeseed, Hubei Hongshan Laboratory, Huazhong Agricultural University, Wuhan 430070, China; National Key Laboratory of Crop Genetic Improvement, National Engineering Research Center of Rapeseed, Hubei Hongshan Laboratory, Huazhong Agricultural University, Wuhan 430070, China; National Key Laboratory of Crop Genetic Improvement, National Engineering Research Center of Rapeseed, Hubei Hongshan Laboratory, Huazhong Agricultural University, Wuhan 430070, China; National Key Laboratory of Crop Genetic Improvement, National Engineering Research Center of Rapeseed, Hubei Hongshan Laboratory, Huazhong Agricultural University, Wuhan 430070, China

## Abstract

Allopolyploids have successfully overcome ‘genome shock’, yet how their subgenomes adapt to coexistence remains largely unclear. Here, we constructed high-resolution epigenomic maps for the diploids *Brassica rapa* (ArAr) and *Brassica oleracea* (CoCo), and examined epigenomic variation in the allotetraploid *Brassica napus* (AnAnCnCn) relative to its putative progenitors. We discovered that coordinated genomic and epigenomic reprogramming in *B. napus* drove convergence of sequence and epigenomic features between An and Cn, significantly reducing expression divergence in homoeologs. Convergent homoeologs were functionally enriched in pathways related to genome stability and abiotic stress responses. Notably, Cn in *B. napus* exhibited greater sequence conservation and epigenetic homeostasis. Furthermore, transcription factor binding sites (TFBSs) affected by genomic variation in An showed convergent regulatory changes toward Cn, indicating that allopolyploids mitigate subgenomic conflicts through multilayered regulatory coordination. In conclusion, coordinated genomic and epigenomic convergence provides critical insights into the stability and adaptive evolution of allopolyploids.

## Introduction

Allopolyploidization is a major evolutionary force in plants, combining divergent genomes through interspecific hybridization and whole-genome duplication to form a more complex genetic architecture [1–3]. Compared to their diploid progenitors, allopolyploids often exhibit enhanced environmental adaptability and domestication potential [4, 5]. These advantages are attributed to the synergistic effects of expanded genomic diversity, reconstructed subgenome interaction networks, and dynamic epigenetic regulation [6–8]. The ‘genome shock’ induced by allopolyploidization leads not only to large-scale chromosomal rearrangements [9–11], but also to widespread epigenomic reprogramming [12–16], including altered histone modifications and changes in accessible chromatin regions (ACRs). Given that the activity of regulatory elements frequently coincides with chromatin state alterations in their vicinity, thereby modulating homoeologous gene expression patterns [17, 18]. However, the relationship between intersubgenomic expression balance and the underlying genomic and epigenomic remodeling in allotetraploids remains poorly understood.

Recent advances have revealed that allotetraploids mitigate conflicts between divergent ancestral genomes through genomic and epigenomic convergence, offering new perspectives on polyploid adaptation mechanisms [14, 19–21]. In vertebrates, allotetraploid species such as carp and crucian carp exhibit structural convergence, rather than divergence, following interspecific hybridization, suggesting that genome architecture can evolve toward compatibility postpolyploidization [20]. Similar patterns have been observed in plants: domesticated allotetraploid cotton (*Gossypium hirsutum*) displays a convergent distribution of DNase I hypersensitive sites (DHSs) that were initially divergent between its diploid progenitors, highlighting chromatin accessibility remodeling as a key mechanism for harmonizing subgenomic expression [19]. At the molecular level, studies on allotetraploid *Arabidopsis suecica* (2*n* = 4*x* = 26, AATT) have shown synchronized DNA methylation changes between subgenomes, where convergent differentially methylated regions (DMRs) are associated with reduced expression divergence of linked genes [14]. Allopolyploids drive subgenome evolution from ‘conflict competition’ to ‘functional complementarity’ through multilayered regulatory strategies, thereby achieving transcriptional balance [22–24]. However, the relationship between genomic and epigenomic convergence and homoeologous gene expression regulation in allopolyploids remains insufficiently characterized.

*Brassica napus* (2*n* = 4*x* = 38, AnAnCnCn), a classical model for allotetraploid species, originated from natural hybridization between *Brassica rapa* (2*n* = 2*x* = 20, ArAr) and *Brassica oleracea* (2*n* = 2*x* = 18, CoCo), followed by whole-genome duplication [25–29]. With well-defined subgenomic origins and frequent homoeologous chromosome exchanges [26, 30], *B. napus* provides an ideal system for investigating polyploid regulatory mechanisms. Notably, the complex epigenetic interplay between An and Cn offers valuable insights into genome coordination and stability [31, 32]. Previous studies have reported subgenomic divergence in chromatin states and functional differentiation of regulatory elements in *B. napus* [33–35]. However, the extent and characteristics of coordinated genomic and epigenomic convergence between its subgenomes remain largely unexplored.

To construct high-resolution epigenomic maps for diploid and allotetraploid *Brassica* species, we generated and integrated datasets encompassing four representative histone modifications, chromatin accessibility, DNA methylation, and transcriptomes. Comparative analyses of *cis*-regulatory divergence between the diploids and the allotetraploid revealed that *B. napus* has established a coordinated mode of genomic and epigenomic convergence, achieved through the remodeling of intersubgenomic architecture and chromatin landscapes. This convergence was associated with a significant reduction in expression divergence between An and Cn. Furthermore, we identified convergence of *cis*-regulatory elements (CREs), wherein transcription factor binding motifs (TFBS) newly acquired in An were linked to enhanced environmental adaptability. Collectively, the high-quality epigenomic resources generated in this study provide a valuable foundation for advancing functional genomics and evolutionary research in the genus *Brassica*.

## Results

### Conservation of epigenetic regulatory features in *B. napus* and its diploid progenitors

**Figure 1:**
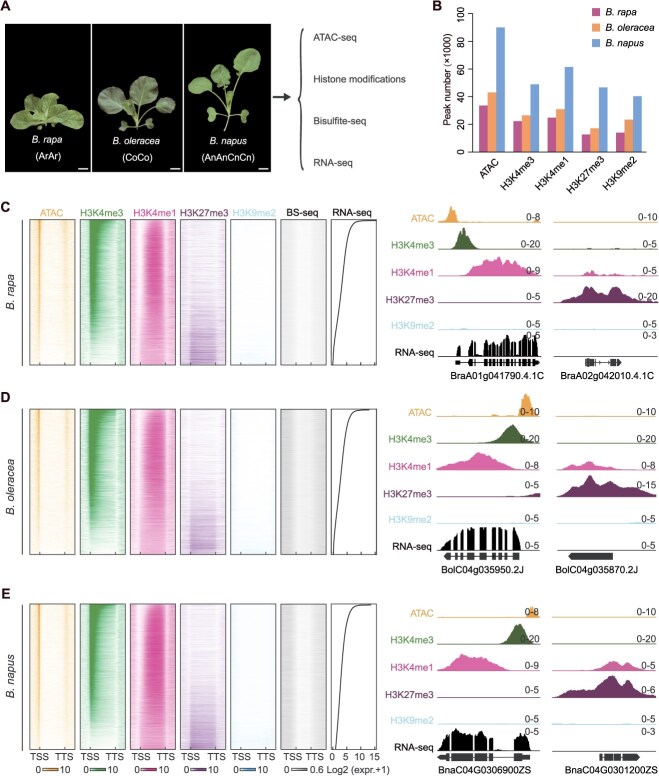
Comparative epigenomic landscapes and transcriptional regulatory features in diploid progenitors and allotetraploid *B. napus*. (A) Phototypes of *B. rapa*, *B. oleracea*, and *B. napus* plants and data generation in this study. Two-week-old plants were shown. Bar, 2 cm. (B) Number of peaks for four histone modifications and ACRs. (C) *B. rapa*, (D) *B. oleracea*, and (E) *B. napus*: Distribution of all epigenomic feature signals near gene regions (±2 kb). Genes were categorized by expression levels. Representative IGV browser examples display a highly expressed gene (left) and a lowly expressed gene (right), along with associated histone modifications and nearby regions of chromatin accessibility.

To systematically construct genome-wide epigenetic regulatory maps for the allotetraploid *B. napus* and its putative diploid progenitors, *B. rapa* and *B. oleracea*, we generated an integrated multi-omics dataset comprising ChIP-seq, ATAC-seq, BS-seq, and RNA-seq (Fig. 1A, Fig. S1, Table S1). These datasets profiled four representative histone modifications (H3K4me3, H3K4me1, H3K27me3, and H3K9me2), genome-wide chromatin accessibility, DNA methylation profiles, and transcriptomic landscapes (Figs S2, S3A and B). To ensure data reliability, only uniquely mapped reads were used for downstream analyses. Pearson’s correlation coefficients between biological replicates exceeded 0.95 (Fig. S1, Table S1). We identified 12 645–33 618 peaks in *B. rapa*, 17 136–39 643 peaks in *B. oleracea*, and 40 308–90 054 peaks in *B. napus*, highlighting a substantial increase in epigenomic complexity following allopolyploidization (Fig. 1B). This increase correlated with genome size differences: the allotetraploid *B. napus* (~961 Mb) possesses a significantly larger genome than its diploid progenitors, *B. rapa* (~426 Mb) and *B. oleracea* (~540 Mb).

Gene expression gradient analyses revealed conserved patterns of epigenetic regulation across diploid and allotetraploid genomes. Activating marks such as H3K4me3 and ACRs were predominantly enriched in the promoters of highly expressed genes, while repressive marks including H3K27me3 and H3K9me2 were associated with genes of low expression (Fig. 1C–E and Fig. S2). Quantitative correlation analyses further confirmed this relationship: H3K4me3 (Pearson’s *r* of 0.74–0.79) and chromatin accessibility (Pearson’s *r* of 0.60–0.66) showed strong positive correlations with gene expression, whereas H3K9me2 displayed a weak but consistent negative correlation (Pearson’s *r* of −0.12 to −0.24) (Fig. S3C). Notably, despite the expansion of epigenetic features in the allotetraploid genome, core regulatory characteristics, including promoter-specific distribution of histone marks and the strength of epigenome-transcriptome coupling, remained largely conserved relative to the diploid progenitors. This finding demonstrates that epigenetic regulatory functions are conserved between diploids and the allotetraploid.

### Asymmetric epigenomic reprogramming of An and Cn in *B. napus*

**Figure 2:**
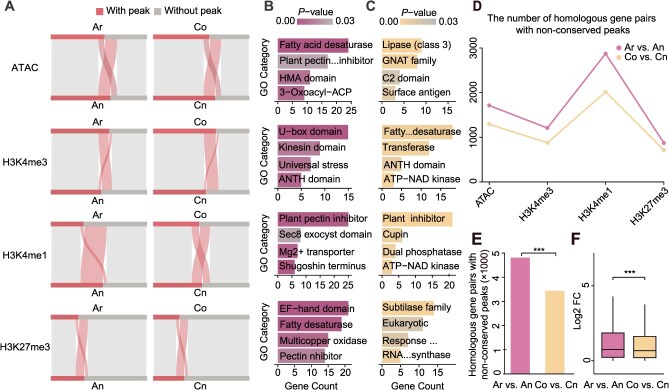
Asymmetric epigenomic reprogramming in subgenomes of allotetraploid *B. napus* relative to its diploid progenitors. (A) Comparison of histone modifications and ACRs between diploid and allotetraploid homoeologous gene pairs. (B) GO enrichment analysis of homoeologous genes with altered histone modifications and chromatin accessibility in Ar vs An. (C) GO enrichment analysis of homoeologous genes with altered histone modifications and chromatin accessibility in Co vs Cn. (D) The number of homoeologous gene pairs with nonconserved peaks in Ar vs An and Co vs Cn. (E) Cumulative number of homoeologous gene pairs with nonconserved epigenomic peaks in Ar vs An and Co vs Cn. (F) Comparison of fold changes in differential expression between homoeologous genes in Ar vs An and Co vs Cn. ^***^P <0.001.

To systematically compared epigenomic changes between diploids and tetraploid, we analyzed 14 748 stringently filtered homoeologous tetrad genes, each exhibiting a strict 1:1:1:1 correspondence across Ar, Co, An, and Cn genomes (Table S2). By comparing the promoter-associated epigenomic features of homoeologous genes between diploid progenitors (Ar, Co) and tetraploid subgenomes (An, Cn), we observed a notable reduction in H3K4me3-marked genes in both An and Cn of *B. napus* (Fig. 2A). Specifically, An showed an 8.0% decrease in H3K4me3-marked genes compared to Ar (Ar: 8970, An: 7788, chi-square test, *P*-value <2.2e−16), while Cn showed a 12.6% reduction relative to Cr (Co: 9733, Cn: 8146, chi-square test, *P*-value <2.2e−16). In contrast, the number of genes marked by the repressive modification H3K27me3 increased by 6.5% in An (Ar: 4190, An: 5142, chi-square test, *P*-value <2.2e−16) and by 5.5% in Cn (Co: 3958, Cn: 4734, chi-square test, *P*-value <2.2e−16). The proportion of homoeologous genes associated with ACRs also increased, by 6.4% in An (Ar: 8290, An: 9238, chi-square test, *P*-value <2.2e−16) and 6.3% in Cn (Co: 8660, Cn: 9588, chi-square test, *P*-value <2.2e−16) (Fig. 2A). These results suggest that the extensive epigenomic reprogramming of *B. napus* subgenomes relative to their diploid progenitors may have been driven by hybridization or by natural variation among cultivars. Gene Ontology (GO) enrichment analyses revealed that epigenetic innovations in An were enriched in core metabolic processes and stress response pathways, suggesting that An contributes to environmental adaptability through dynamic epigenomic regulation (Fig. 2B). In contrast, epigenomic changes in Cn were primarily associated with membrane transport, cytoskeletal organization, and energy metabolism, implicating a role in structural and signaling regulation (Fig. 2C).

To further investigate the coupling of epigenomic and genomic variation, we assessed the conservation of epigenomic features (ACRs, H3K4me3, H3K4me1, H3K27me3) and their associated sequences in the promoter regions of homoeologous gene pairs. In Ar vs An, 55.1%–62.9% of peaks exhibited dual conservation of both sequence and epigenomic features (‘conserved peaks’), slightly lower than the 55.8%–65.0% observed in Co vs Cn (Fig. S4). However, the extent of divergence was greater in An: 32.6% (4809 of 14 748) of Ar vs An homoeologous gene pairs exhibited nonconserved sequence and nonconserved epigenomic features (‘nonconserved peaks’), significantly higher than the 23.3% (3437 of 14 748) observed in Co vs Cn (χ^2^ = 316.4, *P*-value <2.2e−16) (Fig. 2D and E, Fig. S5). Transcriptomic analyses supported this asymmetry: Ar vs An showed a higher proportion of homoeologous gene pairs with large expression divergence (|log2FC| > 1) compared to Co vs Cn (Fig. 2F). Together, these results indicate that allopolyploidization in *B. napus* induced asymmetric epigenomic reprogramming between its subgenomes, with An exhibiting more pronounced innovation. This asymmetry likely serves as a critical driver of adaptive evolution in polyploid plants.

### Chromatin state remodeling reduces transcriptional divergence between An and Cn

**Figure 3:**
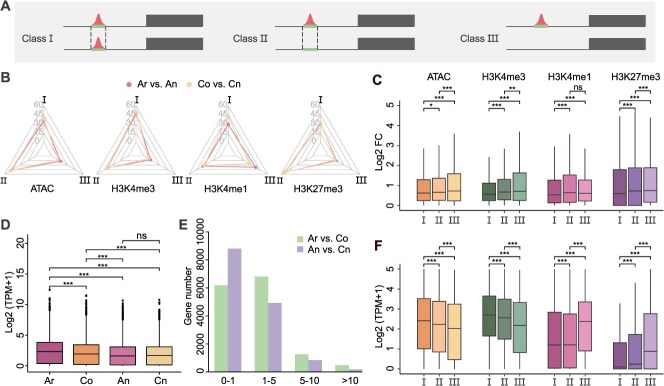
Impact of epigenomic conservation on expression divergence between diploid progenitors and allotetraploid *B. napus*. (A) Three classes of homoeologous gene pairs with different combinations of epigenomic features: Class I: Homoeologous gene pairs with both conserved epigenomic features and conserved sequences. Class II: Homoeologous gene pairs with divergent epigenomic features but conserved sequences. Class III: Homoeologous gene pairs with both divergent epigenomic features and divergent sequence features. Black boxes, gene body; black solid line, promoter region; red peak, epigenomic peaks; green boxes, peak-associated sequences; black dashed line, conserved sequences. (B) Proportions of three classes of homoeologous gene pairs across distinct epigenomic features in Ar vs An and Co vs Cn. (C) Expression fold changes (log2 (max TPM/min TPM)) of three classes of homoeologous gene pairs across different histone modifications and ACRs in Co vs Cn. ^*^P <0.05; ^***^P <0.001; ns, *P* >0.05. (D) Comparison of expression levels of homoeologous tetrad genes in ZS11. (E) Statistics of homoeologous gene expression divergence. Gene expression levels (TPM values) were compared, and the gene number was calculated as the difference in TPM value as follows: 0–1, 1–5, 5–10, and >10. (F) Comparison of gene expression levels among the three gene classes across different histone modifications and ACRs in Co vs Cn.

To investigate the impact of epigenomic remodeling on the expression divergence of homoeologous gene pairs between diploid progenitors and allotetraploid *B. napus*, we categorized 14 748 homoeologous gene pairs into three classes based on the conservation of promoter-associated epigenomic features (histone modifications and chromatin accessibility) and underlying sequence conservation: Class I (homoeologous gene pairs with both conserved epigenomic features and conserved sequences), Class II (homoeologous gene pairs with divergent epigenomic features but conserved sequences), and Class III (homoeologous gene pairs with both divergent epigenomic features and divergent sequences) (Fig. 3A and B). We quantified gene expression divergence using fold-change values between homoeologous gene pairs. Class I gene pairs, which retained both epigenomic and sequence conservation, showed significantly smaller expression divergence than Class II or III, irrespective of whether the epigenomic features were activating or repressive (Fig. 3C, Fig. S6A). Further transcriptome analysis revealed that genes marked by the activating histone modification H3K4me3 in Class I and II exhibited significantly higher expression levels than those in Class III (chi-square test, *P*-value <1e−5), whereas genes with the repressive mark H3K27me3 displayed the opposite trend, with Class III genes showing the highest repression (Fig. 3F, Fig. S6B). These results suggest that epigenomic remodeling, particularly in promoter histone modifications and chromatin accessibility, contributes to the modulation of expression divergence in *B. napus* homoeologs.

To further assess transcriptional changes after polyploidization, we compared expression levels of homoeologous tetrad genes between the diploid progenitors (Ar and Co) and the corresponding subgenomes (An and Cn) in *B. napus* (Fig. 3D, Fig. S7). In both *B. napus* varieties, ZS11 and 2063A, overall expression levels of An and Cn homoeologs were significantly reduced compared to their diploid counterparts Ar and Co (Fig. 3D, Fig. S8). Notably, expression levels of An and Cn also exhibited greater similarity to each other than did Ar and Co, indicating a convergence of transcriptional activity between the subgenomes in *B. napus*. This transcriptional convergence was further supported by comparative analysis of intersubgenomic expression divergence: the diploid progenitors Ar and Co displayed a significantly greater number of highly divergent gene pairs than did An and Cn in the allotetraploid (chi-square test, *P*-value <2.2e−16) (Fig. 3E). Collectively, these findings demonstrate that epigenomic remodeling in *B. napus* is associated with reduced transcriptional divergence between subgenomes, suggesting that intersubgenomic transcriptional convergence is a hallmark of allopolyploid genome stabilization.

### Epigenomic convergence between subgenomes facilitates adaptation in *B. napus*

**Figure 4:**
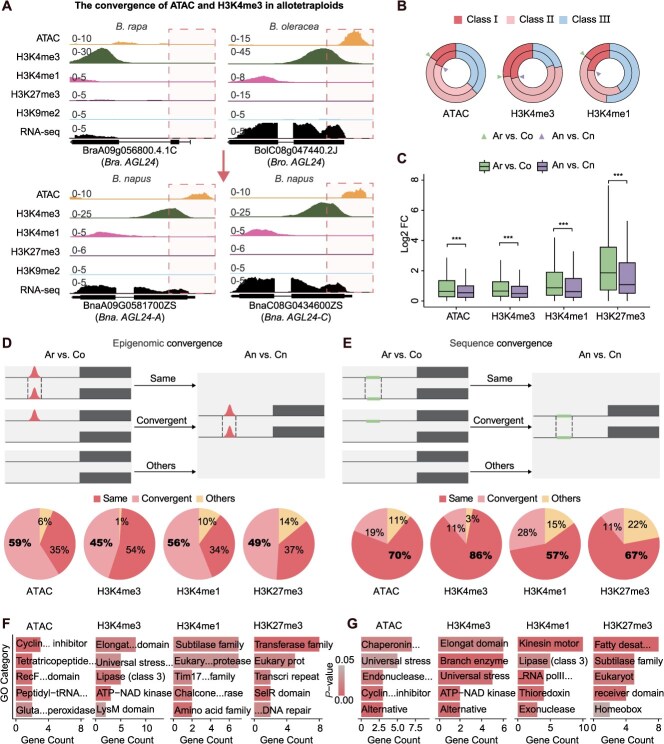
Coordinated genomic and epigenomic convergence between subgenomes of allotetraploid *B. napus*. (A) IGV examples illustrating convergent epigenomic features of ACRs and H3K4me3 in An vs Cn compared with Ar vs Co. (B) Proportions of three classes of homoeologous gene pairs in Ar vs Co (outer circle) and An vs Cn (inner circle) in ZS11, across different histone modifications and ACRs. (C) Comparison of differential expression fold changes for homoeologous tetrad genes with convergent epigenomic features in Ar vs Co and An vs Cn. (D) Schematic diagram of epigenomic convergence. Proportions of three categories among homoeologous gene pairs with conserved epigenomic features in promoter regions of An vs Cn: Same: epigenomic features conserved in Ar vs Co; Convergent: epigenomic features not conserved in Ar vs Co; Others: No peak detected in Ar vs Co. (E) Schematic diagram of sequence convergence. Proportions of three categories among homoeologous gene pairs with conserved sequences in promoter regions of An vs Cn: Same: sequences conserved in Ar vs Co; Convergent: sequences not conserved in Ar vs Co; Others: No peak detected in Ar vs Co. (F) GO enrichment analysis of genes showing convergent changes among homoeologous gene pairs with conserved epigenomic features in promoter regions of An vs Cn. (G) GO enrichment analysis of genes showing convergent changes among homoeologous gene pairs with conserved sequences in promoter regions of An vs Cn.

To test the hypothesis of intersubgenomic epigenomic convergence following polyploidization, we systematically compared the conservation of epigenomic features (including histone modifications and ACRs) in homoeologous tetrad genes between the diploid progenitors (Ar vs Co) and the corresponding subgenomes in *B. napus* (An vs Cn). We found that the proportions of Class I and Class II homoeologous gene pairs in the allotetraploid An vs Cn were significantly higher than those in the diploid Ar vs Co (Fig. 4A and B, Fig. S9). For example, Class I gene pairs marked by H3K4me1 accounted for 22.9% (1911 in 8333) in An vs Cn, significantly higher than 15.0% (1212 in 7873) in Ar vs Co (chi-square test, *P*-value <2.2e−16). Similarly, Class II genes marked by H3K4me1 comprised 35.6% (2967 in 8333) in An vs Cn, compared to 33.0% (2578 in 7873) in Ar vs Co. Class III gene pairs marked by H3K4me1 exhibited the opposite pattern, accounting for 41.5% (3455 in 8333) in An vs Cn and 52.0% (4083 in 7873) in Ar vs Co (Fig. 4B). This convergent pattern was also observed in the 2063A variety (Fig. S10A). These results indicate that coordinated epigenomic and sequence-level convergence occurred between subgenomes in *B. napus* following polyploidization.

Further classification revealed two distinct modes of convergence in *B. napus*: (i) epigenomic convergence, where tetrad genes had divergent epigenomic features in Ar vs Co but showed conservation in An vs Cn; and (ii) sequence convergence, where sequence features were nonconserved in the diploids but became conserved in the tetraploid (Fig. 4D and E). Among An vs Cn, 45%–59% of homoeologous gene pairs with conserved epigenomic features (Class I) showed epigenomic convergence, while 11%–28% of sequence-conserved genes (Class I and II) showed sequence convergence (Fig. 4D and E). Conversely, 34%–54% of genes with conserved epigenomic features in An vs Cn were also conserved in Ar vs Co, while sequence conservation between subgenomes was more extensive (57%–86%) (Fig. 4D and E). These results highlight widespread epigenomic remodeling between the subgenomes of *B. napus*, with genomic sequence features remaining relatively stable. Functional enrichment analysis revealed that genes undergoing epigenomic convergence were significantly enriched in categories related to genome stability, stress response, and post-translational modification pathways (transferase family and HhH-GPD superfamily base excision DNA repair proteins), implicating a role in rapid environmental adaptation via epigenomic plasticity (Fig. 4F). However, genes exhibiting sequence convergence were mainly involved in core metabolic processes, transcriptional regulation, and cellular structure maintenance (U-box domain proteins and alternative oxidases), reflecting functional optimization of essential biological functions (Fig. 4G).

To assess whether the coordinated genomic and epigenomic convergence observed in natural *B. napus* is rapidly established following allopolyploidization, we reanalyzed previously published epigenomic datasets for resynthesized *B. napus* and its diploid progenitors, including H3K27me3 and H3K4me3 modifications [35]. In the resynthesized allotetraploid, the proportion of Class I homoeologous gene pairs was higher in the An vs Cn comparison than in the Ar vs Co comparison (Fig. S10B), with An and Cn expression levels also showing greater similarity than those of Ar and Co (Fig. S10C). Notably, 59%–63% of Class I homoeologous gene pairs in An vs Cn exhibited epigenomic convergence (Fig. S10D) and displayed significantly lower expression divergence than their diploid counterparts (Fig. S10E), supporting a role for epigenomic convergence in transcriptional coordination. Moreover, 113–1919 (16%–36%) of these Class I homoeologous gene pairs overlapped with those identified in ZS11 (Fig. S10F), a proportion significantly greater than expected under a random distribution model. Together, these findings indicate that coordinated genomic and epigenomic convergence can be rapidly established in newly formed allotetraploids.

To further dissect the functional consequences of epigenomic convergence, we analyzed the expression profiles of homoeologous tetrad genes exhibiting conserved epigenomic features. Genes showing epigenomic convergence displayed significantly lower expression divergence in An vs Cn compared to Ar vs Co (Fig. 4C), supporting the role of epigenomic convergence in transcriptional coordination. Among genes with convergent H3K4me3 marks, 57% exhibited expression convergence between An and Cn, a significantly higher proportion than those marked by H3K4me1 (50%) or H3K27me3 (39%) (Figs S11 and S12). Integration with chromatin accessibility data further revealed that H3K4me3-marked genes with expression convergence had higher levels of promoter accessibility than nonconvergent counterparts (Fig. S13). Collectively, these findings indicate that H3K4me3-mediated epigenomic convergence plays a critical role in balancing homoeologous gene expression and may facilitate adaptive evolution in *B. napus* by promoting regulatory compatibility between subgenomes.

**Figure 5:**
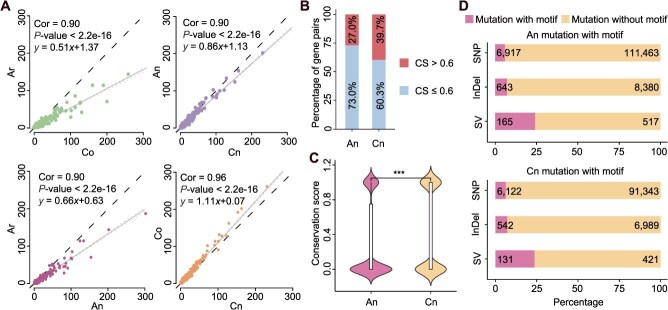
Genomic variation reshapes *cis*-regulatory element landscapes in promoters of *B. napus*. (A) Correlation plot showing the number of genes regulated by TF binding motifs in *x*-axis or *y*-axis. Dashed line: *y* = *x*; line with shading: simple linear regression line with confidence intervals. (B) The stacked plot showed the total proportion of homoeologous gene pairs. (C) Comparison of TFBS conservation score differences between homoeologous gene pairs in An and Cn. (D) Counts of genomic variant types in Ar vs An (top) and Co vs Cn (bottom) comparisons, and proportions of variants overlapping TFBSs in nonconserved ACRs.

### *Cis*-regulatory convergence reflects asymmetric genomic variation in *B. napus*

To investigate the divergence and convergence of *cis*-regulatory elements, we systematically annotated TFBSs in diploid progenitors and allotetraploid (Table S3). A total of 188 179 TFBSs were identified in the promoters of *B. napus*, markedly exceeding those in *B. rapa* (87 468) and *B. oleracea* (115 489) (Tables S4–S6), suggesting that regulatory complexity is substantially enhanced in the allotetraploid relative to its diploid progenitors. To quantify *cis*-regulatory divergence and convergence, we analyzed the number of homoeologous genes with promoter-localized TFBSs across the four (sub)genomes (Ar, Co, An, and Cn). TF motifs were found to regulate a comparable number of genes across all (sub)genomes, with strong correlations observed in each comparison (cor = 0.90, from Ar to Co; cor = 0.90, from An to Cn; cor = 0.90, from Ar to An; cor = 0.96, from Co to Cn) (Fig. 5A). However, global shifts in TF target gene counts revealed a 38.5% increase in An relative to Ar, and a 12.3% decrease in Cn compared to Co (simple linear regression; slope = 0.66, from Ar to An; slope = 1.11, from Co to Cn). Furthermore, the regulatory similarity between An and Cn increased markedly, representing a 68.6% increase compared to the diploid progenitors (simple linear regression; slope = 0.51, from Ar to Co; slope = 0.86, from An to Cn) (Fig. 5A). These results indicate that allopolyploidization promoted intersubgenomic regulatory convergence, likely through the rebalancing of TF–target relationships. Notably, An acquired novel stress-responsive TF binding motifs such as ANAC005 and ERF4/ERF9, which were absent in Ar, potentially contributing to enhanced environmental responsiveness in *B. napus* through the establishment of novel regulatory circuits (Fig. S14). To further assess regulatory element conservation, we calculated conservation scores (CS) for TFBSs in promoter regions (Table S7). Only 27.1% of homoeologous gene pairs in An exhibited high TFBS conservation (CS > 0.6), significantly lower than the 39.8% observed in Cn (Fig. 5B). Moreover, the overall distribution of CS values was significantly higher in Cn than in An (Fig. 5C), indicating asymmetric conservation of *cis*-regulatory elements between subgenomes.

To investigate the underlying causes of TFBS divergence, we scanned for genomic variants in the promoters of homoeologous gene pairs with nonconserved ACRs. In An, we detected 682 structural variations (SVs), 9023 insertions/deletions (InDels), and 118 380 single nucleotide variations (SNVs), all of which exceeded the corresponding counts in Cn (SVs: 552; InDels: 7531; SNVs: 97 465), highlighting greater genomic instability in An. Functional annotation revealed that 23.7%–24.2% of SVs, 7.1%–7.2% of InDels, and 5.8%–6.3% of SNVs overlapped with nonconserved TFBSs (Fig. 5D), indicating that SVs exerted a greater impact on *cis*-regulatory conservation compared to SNVs Subsequently, we investigated the relationship between variation lengths accumulated in promoter regions of homoeologous gene pairs and CS of TFBSs. We further examined how the accumulation of genomic variation in promoter regions affects TFBS conservation. A clear negative correlation was observed between the length of sequence variation and TFBS conservation scores, indicating that extensive genomic variation reduces regulatory conservation (Fig. S15). Collectively, these results demonstrate that *B. napus* underwent *cis*-regulatory innovation driven by asymmetric genomic variation between subgenomes, particularly in An. This regulatory plasticity, facilitated by TFBS turnover and novel motif acquisition, likely contributes to the adaptive potential of the allotetraploid.

## Discussion

Alleviating conflicts arising from distinct parental genomes is essential for the evolutionary success of allopolyploids [20]. In this study, we compared two natural *B. napus* varieties (ZS11 and 2063A) with their putative diploid progenitors (*B. rapa* and *B. oleracea*), and uncovered a pronounced convergence of epigenomic features and associated genomic sequences between An and Cn. This convergence was functionally linked to the transcriptional balancing of homoeologous gene pairs. In addition, CREs also exhibited intersubgenomic convergence, collectively establishing a paradigm of ‘genomic and epigenomic coordinated convergence’. This dual-layered convergence likely mitigates regulatory incompatibilities between subgenomes and contributes to a stable transcriptional regulatory equilibrium in *B. napus*.

The epigenomes of polyploid plants often undergo extensive and systematic reorganization [10, 36–38]. Through comparative analyses with diploid progenitors, we found that epigenomic conservation between subgenomes in allopolyploids was generally lower than that of their underlying genomic sequences. This observation is consistent with the convergent evolution of DNA methylation reported in allopolyploid Arabidopsis [14], suggesting that genome may provide evolutionary anchor points for epigenomic reprogramming, while the flexible nature of epigenomic modifications enables dynamic buffering of inter-subgenomic regulatory conflicts. Notably, *Gossypium* (cotton) achieves homoeologous expression balance through transposon-mediated chromatin remodeling [21, 24], whereas *B. napus* relies on a dual convergence of *cis*-regulatory elements. These species-specific regulatory strategies may be attributed to differences in genome architecture [39, 40] (such as the higher transposon content in cotton) and distinct polyploidization histories, with *B. napus* arising ~7500 years ago, compared to 1–2 million years ago in cotton [26, 41].

Intriguingly, unlike the balanced subgenome evolution observed in some allopolyploid plants [22, 23, 42, 43], *B. napus* exhibited pronounced asymmetry in subgenomic convergence. An displayed greater epigenomic plasticity and more extensive remodeling of regulatory networks, whereas Cn retained higher sequence conservation and epigenomic stability. This asymmetry likely originated from ancestral genomic heterogeneity: the A-genome progenitor (ArAr) harbors expanded clusters of tandemly duplicated genes (a 1.8-fold increase in disease-resistant NBS-LRR genes) potentially contributing to regulatory redundancy and adaptive flexibility. In contrast, the C-genome progenitor (CoCo) preserved conserved, single-copy gene networks associated with essential metabolic pathways (brassinosteroid biosynthesis) [44, 45]. These findings suggest that coordinated genome evolution in allopolyploids is shaped not only by epigenomic plasticity but also by a preexisting hierarchical regulatory architecture embedded within the ancestral genomes.

The ‘coordinated genomic and epigenomic convergence’ framework proposed in this study offers a novel perspective on the evolutionary dynamics of allopolyploids. Future research should focus on: (i) deciphering the spatiotemporal coupling of genomic and epigenomic convergence during the early stages of polyploidization; (ii) investigating the role of 3D genome architecture in mediating intersubgenomic coordination; (iii) elucidating the mechanisms by which epigenetic memory is stabilized under multigenerational selection. Collectively, these insights not only advance our understanding of polyploid genome evolution but also establish a theoretical foundation for precision breeding of polyploid crops.

## Conclusion

In summary, we generated the most comprehensive and high-quality epigenomic maps for the diploid species *B. rapa* and *B. oleracea*, encompassing four representative histone modifications, chromatin accessibility, DNA methylation, and transcriptome data. Comparative genomic analyses revealed that coordinated genomic and epigenomic convergence plays a critical role in maintaining transcriptional balance between subgenomes in the allotetraploid *B. napus*. This pattern was consistently observed across two distinct *B. napus* varieties (ZS11 and 2063A). Notably, *B. napus* exhibited asymmetric subgenomic convergence, with Cn displaying stronger sequence conservation and epigenetic stability. While genomic sequences were generally more stable than epigenomic features, *cis*-regulatory elements also showed convergent evolution across subgenomes. Together, these findings highlight that coordinated genomic and epigenomic convergence serves as a fundamental mechanism enhancing the regulatory robustness and adaptive potential of *B. napus*.

## Materials and methods

### Plant materials and growth conditions

In this study, young leaves from *B. rapa* (Chiifu) and *B. oleracea* (JZS) and *B. napus* (ZS11 and 2063A) [31, 46] were analyzed. For the collection of young leaves, germinated seeds were grown in the phytotron with a day/night cycle of 16 h/8 h and a temperature of 22°C/18°C. Young leaves were obtained from 2-week-old plants cultured hydroponically.

### ChIP-seq library preparation

The ChIP-seq libraries were constructed according to the previous study [31, 47]. Young leaves were collected from 2-week-old hydroponically cultured plants grown under controlled phytotron conditions (16 h light/8 h dark cycle; 22°C/18°C), as previously described [48]. Fresh leaves were cross-linked with 1% formaldehyde and quenched with 0.2 M glycine. For each ChIP-seq experiment, 0.5 g of tissue was cryogenically ground in liquid nitrogen to a fine powder and lysed in 1.5× volume of buffer S (50 mM HEPES-KOH, 150 mM NaCl, 1 mM EDTA, 1% Triton X-100, 0.1% sodium deoxycholate, 1% SDS, 1× protease inhibitor cocktail) for 15 min with rotation. Samples were then diluted with 6× volume of buffer F (buffer S without SDS), and chromatin was fragmented using a Bioruptor (Diagenode). After centrifugation (12 000× g, 10 min, 4°C), supernatants were transferred to fresh tubes for immunoprecipitation.

ChIP samples were incubated with 5 μg of antibodies targeting H3K4me1 (ABclonal, A2355), H3K4me3 (ABclonal, A2357), H3K9me2 (Abcam, ab1220), or H3K27me3 (ABclonal, A2363) overnight at 4°C with rotation. Following reverse cross-linking, DNA was extracted using phenol:chloroform:isoamyl alcohol (25:24:1), ethanol-precipitated, and resuspended in TE buffer. Libraries were constructed using the NEBNext Ultra II DNA Library Prep Kit for Illumina and sequenced on an Illumina HiSeq X Ten system (paired-end 150-bp reads). Two biological replicates were analyzed per experiment.

### ATAC-seq library preparation

The ATAC-seq libraries were constructed according to the previous study [49]. Approximately 0.5 g of young leaves were used per ATAC-seq experiment. Nuclei were isolated by chopping tissues in ice-cold 1× PBS (with 1× protease inhibitor cocktail) using a sterile blade. The homogenate was filtered through Miracloth, and nuclei were pelleted by centrifugation. Approximately 50 000 nuclei were incubated with 40 μl Tn5 transposase mixture (VAHTS TD501; 8 μl TTBL, 4 μl TTE mix, 28 μl H2O) at 37°C for 30 min with 300 rpm rotation. DNA was purified using the Qiagen MinElute Kit, and libraries were amplified for 9–11 cycles. Libraries were size-selected and sequenced (2 × 150 bp) on an Illumina HiSeq X-Ten system.

### Whole-genome bisulfite sequencing library preparation

The whole genomic DNA was extracted using the DNeasy Plant Mini Kit (QIAGEN, 69104). Bisulfite conversion of the DNA was conducted using the EpiTect® Plus DNA Bisulfite kit (QIAGEN, 59124), and the bisulfite-treated DNA libraries were constructed using the Illumina TruSeq DNA sample prep kit. For each BS-seq experiment, 1 μg of DNA was fragmented by sonication in the Bioruptor. The fragmented DNA was processed by end repair, 3′-end adenylation, and adapter ligation. The adapter-ligated DNA was then treated with bisulfite, followed by 9 cycles of polymerase chain reaction (PCR) amplification. Finally, the PCR product was purified using Vazyme clean beads. The libraries were sequenced on an Illumina HiSeq X Ten system (paired-end 150-bp reads).

### RNA-seq library preparation

Total RNA from young leaves was isolated using the RNeasy Plant Mini Kit (QIAGEN, 74904). Approximately 2 μg of RNA was used for library preparation with the Illumina TruSeq RNA kit following the manufacturer’s protocol. The libraries were sequenced on an Illumina HiSeq X-Ten system.

### RNA-seq analysis

RNA-seq reads (150 bp paired-end) were processed using Fastp (v0.22.0) [50] to remove adapters and retain high-quality reads. Filtered reads were aligned to their respective reference genomes (*B. rapa*, *B. oleracea*, and *B. napus*) using BWA mem [27, 28, 51, 52]. Low-quality alignments (MAPQ <30) were filtered with SAMtools (v1.9; samtools view -q 30) [53]. Transcript abundance was quantified as TPM (Transcripts Per Million) using StringTie (v1.3.0) [54]. For differential expression analysis, read counts were generated by featureCounts [55], and differentially expressed genes (DEGs) were identified using DESeq2 [56] in R with thresholds of *P* <0.05 and |log2(fold change)| > 1.

### ChIP-seq analysis

ChIP-seq reads (150 bp paired-end) were trimmed with Fastp (v0.22.0) [50] and aligned to reference genomes using BWA mem. Mitochondrial and chloroplast DNA alignments were removed with SAMtools (v1.9) [53], and PCR duplicates were eliminated using GATK MarkDuplicates (v4.2.3.0) [57]. High-quality alignments (MAPQ ≥30) were retained. Peaks were called using MACS2 (v2.1.1) [58] with parameters -f BAMPE -B -q 1e−5. For H3K9me2, broad peaks were identified using --broad -f BAMPE -B -q 1e−5. FRiP (Fraction of Reads in Peaks) was calculated to assess data quality.

### ATAC-seq analysis

ATAC-seq reads (150 bp paired-end) were processed similarly to ChIP-seq: trimmed with Fastp (v0.22.0) [50], aligned using BWA mem, filtered for MAPQ ≥30, and deduplicated with GATK MarkDuplicates. Peaks were called using MACS2 (v2.1.1) [58] with parameters -f BAMPE -B -q 0.01, and FRiP scores were computed for quality assessment.

### Characterization of chromatin states and assignment of genomic features

We used ChromHMM (v1.12) [59] to determine the significance of the combinatorial information for the different histone marks. For all histone marks (H3K4me3, H3K4me1, H3K27me3, and H3K9me2) and ATAC, read counts were computed in nonoverlapping 200-bp bins across the whole genome. We trained an eight-state model for subsequent analyses, it captured all of the key information between the histone marks.

### DNA methylation analysis

Whole-genome bisulfite sequencing (WGBS) data were evaluated using Fastp (v0.22.0) [50], then filtered the adapter and low-quality reads. The clean reads were mapped to the ZS11 genome [51] using BatMeth2 [60].

### Identification of homoeologous tetrad genes

Orthologous gene pairs between diploids and tetraploids were identified by integrating MCScan [61] and OrthoFinder [62]. OrthoFinder (v2.3.8) was used to analyze proteomes of *B. rapa*, *B. oleracea*, and the two subgenomes of *B. napus*, followed by extraction of 1:1:1:1 orthologs. MCScan identified syntenic blocks with parameter --cscore = 0.99. Only orthologs within syntenic blocks were retained as homoeologous tetrad genes, yielding 14 748 homoeologous tetrad genes where each gene had a strict 1:1:1:1 correspondence across Ar, Co, An, and Cn (sub)genomes.

### Mapping histone modification sites and ACRs to homoeologous genes

The promoter regions (1.5 kb upstream to TSS and 0.5 kb downstream of TSS) of homoeologous tetrad gene were defined as syntenic blocks. Pairwise alignment of syntenic blocks was performed using LASTZ (v1.4.3) with parameters: --notransition --step = 20 --strand = both --format = lav --allocate:traceback = 200 M --hspthresh = 8000 --nogapped. Subsequently, UCSC Genome Browser utilities (chainPreNet, chainNet, and netChainSubset) were sequentially applied to refine the block alignment files. Finally, We used CrossMap (v0.5.4) [63] to extract positions of regions homologous to epigenetic modifications and ACRs from optimized block alignment files.

### Motif analysis

Promoter sequences were extracted from the *B. rapa*, *B. oleracea*, and *B. napus* genomes, followed by independent identification of TFBS. The findMotifs.pl script in HOMER (v4.11.1) [64] was used to detect TF motifs, retaining only hits with motif scores >10. The analysis utilized HOMER’s built-in plant TF motif database containing 506 motifs.

### Calculation of TFBS conservation score

For all TFBSs located in the promoters of orthologous promoter pairs, a TFBS was defined as conserved if it was identified in both diploid and tetraploid homologs. Nonconserved TFBSs were those detected only in tetraploids. CS for each tetraploid gene was calculated as (counts of conserved TFBSs/counts of total TFBSs) × 100%.

### Genomic variation analysis

Promoter sequences of orthologous gene pairs were aligned using MUSCLE (v5.1) [65]. Sequence variants were classified as: SNVs: Base substitutions. InDels: Sequences ≤50 bp in length. SVs: Sequences >50 bp.

## Supplementary Material

Web_Material_uhaf266
