# Supplementary material for: Genomic and epigenomic coordination maintains subgenome transcriptional balance in allotetraploid Brassica napus
Source: Hortic Res. 2025 Oct 3;13(1):uhaf266. doi: 10.1093/hr/uhaf266 (PMC12861482; doi:10.1093/hr/uhaf266)
Supplement: Web_Material_uhaf266 [file web_material_uhaf266.zip › Supplementary Figures.doc]

**Figure S1. Correlation analysis for datasets of *B. rapa* and *B.oleracea*.** Genome-wide correlation heat map (with bin size 500 kb) for ChIP-Seq and RNA-Seq data across young leaves from *B. rapa* and *B.oleracea.*

**Figure S2. Chromatin state definitions, abbreviations and composition (emission probability) of histone marks. Genome coverage and genomic annotation enrichment. Expression levels of genes associated with each chromatin state.**

**A.** Chromatin states in *B. rapa.*

**B.** Chromatin states in *B. oleracea*.

**Figure S3. The characteristics of four histone modifications and chromatin accessibility among *B. rapa*, *B. oleracea*, and *B. napus*, and their relationships with gene expression.**

**A.** The distributions of the four histone modifications and chromatin accessibility near gene regions in *B. rapa*, *B. oleracea*, and *B. napus*.

**B.** The odds ratio across three genomic regions in *B. rapa*, *B. oleracea*, and *B. napus*.

**C.** Correlation between epigenomic features and gene expression levels in *B. rapa*, *B. oleracea*, and *B. napus*.

**Figure S4. Proportions of the three types of peaks in Ar vs. An and Co vs. Cn.** Peaks were classified into three categories: (a) conserved peaks; (b) non-conserved peaks with conserved sequences; (c) non-conserved peaks.

**Figure S5.** **The overlap of homoeologous gene pairs with non-conserved peaks across different histone modifications and ACRs.**

**A.** Ar vs. An; **B.** Co vs. Cn.

**Figure S6. Comparison of expression fold changes and expression levels among three classes of homoeologous gene pairs.**

**A.** Expression fold changes (log2(max TPM/min TPM)) of three classes of homoeologous gene pairs across different histone modifications and ACRs in Ar vs. An. *, *P*-value < 0.05; ***, *P*-value < 0.001; ns, *P*-value > 0.05.

**B.** Comparison of gene expression levels among the three gene classes across different histone modifications and ACRs in Ar vs. An.

**Figure S7. Volcano plots showing fold changes in differential expression of homoeologous gene pairs.**

**A.** Differential expression between Ar and An.

**B.** Differential expression between Co and Cn.

**Figure S8. Comparison of expression levels of homoeologous tetrad genes in 2063A.**

**Figure S9. Proportions of three classes of homoeologous gene pairs in Ar vs. Co and An vs. Cn (ZS11), across different histone modifications and ACRs.**

**Figure S10. Proportions of three classes of homoeologous gene pairs in Ar vs. Co and An vs. Cn in 2063A and resynthesised *B. napus*.**

**A.** Proportions of three classes of homoeologous gene pairs in Ar vs. Co (outer circle) and An vs. Cn (inner circle) in 2063A, across different histone modifications and ACRs.
**B.** Epigenomic data from the reanalysis of resynthesized *B. napus* and its diploid progenitors, *B. oleracea* and *B. rapa*.
**C.** Comparison of expression levels of homoeologous tetrad genes between resynthesized *B. napus* and its diploid progenitors, *B. oleracea* and *B. rapa*.
**D.** Proportions of three categories among homoeologous gene pairs with conserved epigenomic features in promoter regions of An vs. Cn in resynthesized *B. napus*.
**E.** Comparison of differential expression fold changes for homoeologous tetrad genes with convergent epigenomic features in resynthesized *B. napus*.
**F.** Comparison of expected and observed proportions of Class I homoeologous gene pairs in resynthesized *B. napus* that overlap with Class I homoeologous gene pairs in ZS11.

**Figure S11. Proportion of homoeologous tetrad genes exhibiting both convergent expression and convergent epigenomic features.**

**Figure S12. Proportions of homoeologous tetrad genes with convergent versus divergent expression levels among those exhibiting convergent epigenomic features across different histone modifications and ACRs.**

**Figure S13. Differences in ATAC signals near genes with convergent versus divergent expression levels among homoeologous genes exhibiting convergent H3K4me3 marks in *B. napus*.**

**Figure S14. Diagrams showing examples of TF motifs.**

**Figure S15. Correlation between total conservation scores and genomic variation lengths of homoeologous gene pairs in *B. napus*.**

**A.** Gene pairs in An.

**B.** Gene pairs in Cn.
